# Comment on “Assessing Chat Generative Pretrained Transformer- 4’s clinical utility in acute compartment syndrome: a comprehensive evaluation of accuracy, completeness, and readability”

**DOI:** 10.1590/1806-9282.20252268

**Published:** 2026-07-24

**Authors:** Hinpetch Daungsupawong, Viroj Wiwanitkit

**Affiliations:** 1 Private Academic Consultant – Phônhông, Lao People’s Democratic Republic.; 2 Saveetha University, Saveetha Institute of Medical and Technical Sciences, Saveetha Dental College and Hospitals, Department of Research Analytics – Chennai, India.

Dear Editor,

We would like to comment on “Assessing Chat Generative Pretrained Transformer-4’s clinical utility in acute compartment syndrome: a comprehensive evaluation of accuracy, completeness, and readability^1^.” This work is noteworthy because it evaluates the capabilities of advanced artificial intelligence, such as ChatGPT-4, in the setting of high-risk orthopedic surgery emergencies like acute compartment syndrome. However, the research technique has some important shortcomings. First, using only one set of questions from the 2019 American Academy of Orthopaedic Surgeons guidelines may result in positive bias in the evaluation results, as the linguistic model is typically trained on similar data, reflecting “conceptual rote memorization” rather than actual clinical reasoning. Second, the evaluators were limited to two expert physicians, who, despite reflecting Cohen’s kappa ratings, are prone to expert bias and do not represent the opinions of other user groups, such as general practitioners, emergency physicians, or patients.

In terms of statistics and evaluation, reports of up to 95% accuracy for both open-ended and binary questions may be overstated due to a lack of examination of the complexity of the questions or decision-making circumstances under ambiguity. The stated average completeness (2.9±0.5) lacks semantic clarity compared to the whole scale and is not connected with specific therapeutic outcomes. Furthermore, while the DISCERN score is deemed “excellent,” the acknowledgement of poor source trustworthiness reveals structural flaws in the language model. The research lacks the transparency to cite primary sources.

This study may not show that ChatGPT-4 is “ready to use” to support clinical decision-making for acute compartment syndrome, but rather emphasizes its more appropriate role as a cognitive scaffold for healthcare professionals, particularly in reviewing principles, differential diagnosis, and warning of red flags, rather than directly making treatment decisions. The system’s difficulty to read (extremely low Flesch-Kincaid score) indicates that it is implicitly meant for experts rather than patients, which could be both a constraint and an opportunity if a language-adapted version is developed for different target groups.

An important point for a broader discussion is whether medical artificial intelligence (AI) should be judged only on “content accuracy,” or also on its capacity to prioritize, manage ambiguity, and communicate risk. Future studies should use clinical vignettes with incomplete and time-constrained data to compare AI-assisted versus non-AI-assisted physician decision-making. Furthermore, ethical and policy concerns should be raised about the scope of AI use in crises in order to avoid over-reliance on the technology in situations where incorrect decision-making could result in permanent injury or death.

## AI DECLARATION

The authors used language editing computational tool in preparation of the article.

## Data Availability

The datasets generated and/or analyzed during the current study are available from the corresponding author upon reasonable request.
